# Practical support aids addiction recovery: the positive identity model of change

**DOI:** 10.1186/1471-244X-13-201

**Published:** 2013-07-31

**Authors:** Ayna B Johansen, Håvar Brendryen, Farnad J Darnell, Dag K Wennesland

**Affiliations:** 1 Norwegian Centre for addiction Research, Ullevål University Hospital, Postboks 1039, Blindern, 0315 Oslo, Norway; 2 Centre for the Study of Mind in Nature, University of Oslo, PO box 1020 Blindern, N-0316 Oslo, Norway

**Keywords:** Principles of recovery, Grounded theory, Positive identity, Social support

## Abstract

**Background:**

There is a need for studies that can highlight principles of addiction recovery. Because social relationships are involved in all change processes, understanding how social motivations affect the recovery process is vital to guide support programs.

**Methods:**

The objective was to develop a model of recovery by examining addicted individuals’ social motivations through longitudinal assessment of non-professional support dyads. A qualitative, longitudinal study design was used, combining focus groups and in-depth interviews with addicted individuals and their sponsors. Data were analyzed using the principles of grounded theory: open coding and memos for conceptual labelling, axial coding for category building, and selective coding for theory building. The setting was an addiction recovery social support program in Oslo, Norway. The informants included nine adults affected by addiction, six sponsors, and the program coordinator. The participants were addicted to either alcohol (2), benzodiazepines (1), pain killers (1) or polydrug-use (5). The sponsors were unpaid, and had no history of addiction problems.

**Results:**

Support perceived to be ineffective emerged in dyads with no operationalized goal, and high emotional availability with low degree of practical support. Support perceived to be effective was signified by the sponsor attending to power imbalance and the addict coming into position to help others and feel useful.

**Conclusions:**

The findings appear best understood as a positive identity-model of recovery, indicated by the pursuit of skill building relevant to a non-drug using identity, and enabled by the on-going availability of instrumental support. This produced situations where role reversals were made possible, leading to increased self-esteem. Social support programs should be based on a positive identity-model of recovery that enable the building of a life-sustainable identity.

## Background

We need increased insight into recovery principles to help more people overcome their addictions. The long-term recovery rate among people receiving addiction treatment is only incremental to the recovery rate of addicted persons who go untreated [1], suggesting that the theoretical bases of current treatments are inadequate. Specifically, we need to develop theories for how people recovering from addictions progress; [2-5]. Such theories should encompass the longitudinal, social, and interactive processes involved in recovery. To achieve this, one must study how individuals interact with their available life options.

### The dynamic nature of recovery

Because social relationships are involved in motivating drug use [6], understanding how they affect motivation for recovery is vital to guide support programs. From the perspective of the recovered addict, receiving help from supportive people has been identified as the most important factor to one’s personal recovery process [7]. Interventions that include family therapy have been found more effective than individual or mixed approaches [8], and for disenfranchised persons (e.g. those recovering from severe heroin addiction), recovery from the social disruptions caused by the abuse is a simultaneous process, only rivaled in importance by quitting the drug [9]. A review on the effect of Alcoholics Anonymous (AA), a recovery support network, concludes that social support is a mechanism in its effectiveness [10]. Likewise, social capital, or aspects of one's relationships that facilitate action, help mitigate the problems of misuse and aid natural recovery [11,12]. Also, people who are part of non-substance abusing networks, and those who indicate they receive social- and abstinence-support during the time of treatment experience better-treatment outcomes [13]. Moreover, one study has shown that people who became members of a social recovery network achieved abstinence rates at higher rates than those who sought professional help [14]. Indeed, claims are made that the construct of “recovery”, is the very paradigm or culture that enables its’ own phenomenon or existence [15,16].

One of the main reasons why professional treatment works, according to clients, is because it enables more open communication about personal needs with people in positions to be supportive, such as friends and family [17]. People enjoy sharing in support relationships, are motivated by achieving goals, and use the structure of treatment to keep busy [5,18]. Thus, providing people with social opportunities should help facilitate recovery. However, many synthesized or arranged support relationships do not become meaningful to the involved parties, and are either perfunctory or tend to focus on the structure of the support program [19]. Also, some social support interventions have been shown to not relate to recovery or quit rates [20,21], or may not work for all populations [22]. Moreover, the success of the intervention does not relate to some of the individual factors traditionally important to relationship functioning, including avoidant or anxious attachment, anxiety, or depression [23-25]. Making an impact on their effectiveness is thus likely not possible with current matching strategies. Specifically, we need a theory about individual and contextual differences in relation to the relationship processes that make support dyads effective. Therefore, in this study we set out to theorize on addiction recovery collecting qualitative data longitudinally, within synthesized support dyads between people in treatment and supportive community volunteers.

## Methods

A longitudinal grounded theory qualitative research design, including methods- and source triangulation features [26], was utilized to capture the recovery process from multiple angles and within a social context [27]. The methods chosen included focus groups and interviews; the focus groups were conducted first in order to engage the informants and prime reflection prior to the individual in-depth interviews [28]. The study was approved by the Norwegian Regional Ethics Committee, and all people with addictions provided written informed consent, after given time to consider it. No compensation was given.

### Sample

A total of 9 adult people with addictions (4 men, 5 women) were recruited from a social support program for people with substance abuse problems. These individuals had attended the program for an average length of 7 months at the time of recruitment, had varied backgrounds and drugs of choice. Five reported concurrent diagnoses including bipolar disorder, social anxiety, post-traumatic stress disorder, and general anxiety, six had a history of employment problems, and all received simultaneous outpatient treatment. Among the group of sponsors, there were a total of 6 adults (5 women/1 man) of varied backgrounds who had been recruited to the support program based on personal motivation and capacity for volunteering. Finally, the sample included the project coordinator who served as a key informant within the study.

### Setting

The study was conducted at a social support program facilitated by a non-profit organization. The physical locality of the program examined in this study was in close proximity to outpatient treatment-, daytime activity-, and halfway housing facilities, all parts of the same organization. Users of the social support program examined were members or had patient status in one or more of the facilities within the organization.

### Data collection and procedure

The study was planned in three phases over 2.5 years (January 2010 - June 2012). Recruitment was conducted from November 2009 through January 2010. After providing written consent, participants with addictions and sponsors were enrolled into separate focus groups (phase 1), beginning in January 2010. Next, in-depth interviews and validation checks (phase 2) were completed. Validation checks were completed at 9 months (approximately December 2010) by phone interviews for the participants with addictions and a separate focus group for the sponsors. Finally, follow-up interviews (phase 3) started in March 2012 and ended in June 2012. Focus groups and interviews were digitally voice-recorded.

As the coding process unfolded, the key informant was used as part of a reflexive validation process to verify and obtain additional information on the continued contact patterns of the dyads. Multiple conversations took place as part of the process, including a total of three face-to-face meetings, as well as five phone interviews that each lasted half-an-hour or less. The baseline interview was recorded and transcribed, while the other conversations were recorded in notes form only. These conversations focused on the nature and outcome of any individual or dyadic follow-up the key informant had had since the last contact.

### Focus groups

Focus groups were conducted separately for participants with addictions and sponsors so the informants’ common experiences could be explored, and to ensure that people felt safe to express their opinions so to stimulate each others’ responses [29,30]. A graduate student of clinical psychology co-administered the session by recording and taking observational notes. Each of the focus group sessions lasted approximately 2 hours and gave clues to a number of findings.

### Interviews

The focus group interview guides were based on knowledge about program features gathered during preliminary meetings with the key informant. The individual interview guides were based on the concept of therapeutic alliance, targeting displays of passive and active alliance rupture markers [31] to understand the level of safety within the sponsor relationships. Specifically, the interviewer would make sure to include events that may have caused conflict and efforts to resolve said conflicts. With the exception of including past drug-use history and treatment and quitting efforts with the participant individual interviews, the interview guides were identical to those of the sponsors (see Additional file 1 for Interview Protocols). The in-depth interviews were conducted at the program site, while the follow-up interviews were conducted at the program site, or another sheltered location.

### Data analysis

**Figure 1:**
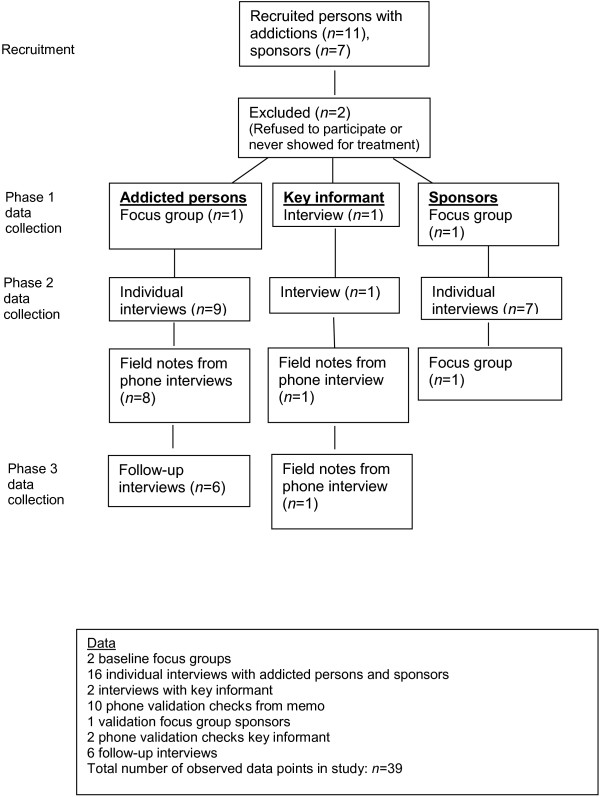
Study data flow of participant contact points.

Prior to analyses all the in-depth interviews were transcribed verbatim. In order to more easily distinguish between relevant and irrelevant content in the later analytical process, the text was transferred and organized in NVivo software. For the analyses, we relied on the constant comparative method, memoing, and axial and theoretical coding techniques. That is, we compared the categories with all incoming data obtained through the baseline interviews. In keeping with the constant comparative method, we analyzed each new datum using the strategies described by Charmaz [24], including open, axial and theoretical coding. In all, a total of *n* = 39 data points (see Figure 1) were registered as part of the study.

Using a full-version [32] and longitudinal grounded theory approach, we first conducted an open coding of the data from phase 1 and phase 2. Next, we used the framework analysis method to track changes over time [33], and facilitate axial coding and constant comparison. Relationships between the codes were explored throughout all three phases of the study and individual changes were covaried with dyadic events and events involving relationships with other people representing network support for either using or non-using. As such, narrative analyses were conducted for all dyads to capture details about the support process and its consequence for recovery. In this way, we were able to describe the support dynamics of each dyad, explore how the support was influenced by characteristics of the individual members and support arrangement, and theorize about the ways this affected recovery. In addition to the tracking of thematic changes, we also utilized proportions as indicators of change [34] p. 233.

## Results

### Focus group first impressions

In reviewing the data from the initial focus groups we found that, all sponsors supported one another, but variations were nonetheless found in approaches to sponsoring, particularly pertaining to focusing on the *use* of substances, as opposed to other outcomes. Likewise, all those affected by addiction recognized fear of stigma, but the group revealed inconsistencies in reporting between the dyadic members, which gave clues to later understanding of sponsoring contributions to ineffective support.

### Longitudinal outcomes

In reviewing the progression of the 9 individuals who had addiction at the 2-year mark, two were judged to be “in recovery” (e.g. only minor lapses (<week) in past year), three were judged to be “improved” (e.g. taken control/improved self-esteem within the past 6 months), three were “unchanged” (e.g. major lapses (>week) in past year/no change in control). Finally, one person had “relapsed.” The core recovery construct (final model) was self-verification of positive identity (pride in socially acknowledged role). Five recovery related process themes were identified in total: (1) positive identity-building activity, (2) support perceived as ineffective (enmeshment), (3) support perceived as effective (mindful management of power imbalance), (4) taking control, and (5) self-verification of positive identity.

When I heard there was a sponsor program, I had some things I knew I immediately needed help with. Specifically, it was computers… and I was looking for a person who knew a specific computer programming. It turned out, that need was so specific, it was too specific for him (the sponsor) to do. However, he had some knowledge of similar programs, and was able to help me with a lot of the organizing that I needed for school. I had managed to get by with my schooling up until that point without any real computer skills… so I got the help I needed from him to get started on a lot of these things. So in our first meetings, we talked and did a lot of things related to that. But we turned into good friends. And I was invited to his cottage, and even though I didn’t go, it all went really well.

Recovered1, follow-up, p.3

1. *Positive identity-building activity:* Successful recovery (the cases of *“Recovered1”* and *“Recovered2”*) was linked with having support relationships that focused on a personally defined growth project (as opposed to the conversation). This was furthermore linked with a change in social status, increase in self-esteem, and internalization of responsibility for substance use. The successful support in these instances was instrumental as well as emotional and the common project was recognized by an emphasis on learning or skill building. Being recovered, in both cases, meant being recognized as improved by themselves, their sponsors, as well as the other addicted participants. For neither person it meant total abstinence, but rather improved self-esteem and confidence in their ability to refrain from using. Reflecting back, *“Recovered1”* attributed much of his success to being able to focus on a shared interest with his sponsor, namely computers:

For *“Recovered2”*, the process of building positive identity was also evident. However, in contrast with *“Recovered1”*, who only “dabbled” in the recovery community and otherwise was largely focused on his education, *“Recovered2”* relies on multiple connections in the community and uses the program with great frequency. In fact, he did not only stop using drugs, but effectively adapted his social life to revolve around recovery-related activities. Encouraged by his sponsor and building confidence, he also started sponsoring someone else. To him, recovery first and foremost meant honesty and pride in his work. Recovery was thus a *self*-recovery more than it was quitting drinking. During the follow-up interview he reflected back on his process:

“Recovered2”: Yes and I was able to maintain a sense of competence all the way, even if I wasn’t too sure all the time, I got positive feedback from Jack… and things never seemed to move faster than I could handle. I’ve had a number of setbacks, but they didn’t last more than a few days, and I usually had them during the weekend. So on Mondays I returned. I’d decided this was where I was going to be (indicating recovery community)

I: So what words do you use to describe yourself now?

“Recovered2”: “I”m an addict. Current. And I’ll always stay that way, but it doesn’t mean I have to drink. And I’m not an ex-addict. I’m just dried up. The second you distance yourself from the addiction you’ve lost. Once an addict, always an addict.

“Recovered2”, follow-up, p. 8

2. *Support perceived to be ineffective: enmeshment:* One important pattern that emerged from the data was the tendency for the support person to worry excessively about the person affected by addiction and the amount of drugs they consumed. As the enmeshment emerged, it was found as a likely consequence of the dyad not having successfully defined a personal project that provided a means for mutual activity., and while one pair sat still while conversing, the other conversed while walking. This pattern only concerned the support relationships of those people with addictions whose dyad activities were centered on the pair itself (e.g. meeting in café, talking while walking), rather than on some other activity (e.g. meeting to take computer to repair shop or to research new art ideas). The pattern was exemplified in people who seemed otherwise quite different; ranging from quite assertive to reluctant to set boundaries, but it was particularly prevalent with two women who both abused prescription drugs. A problem faced by all the sponsors was the frustration of “never knowing how much they have taken.” The value tied to their evaluation of their own support was tied directly to the idea of how much the participant affected by addiction had used on the day of meeting, or days prior to. These thoughts were then connected with verbal confrontation, as in the dyad evolving quickly with “*Improved2”* and her sponsor, and more gradually with *“Unchanged1”* and her sponsor. In both instances, however, the lack of trust in the person affected by addiction reduced the sponsors’ appraisal of the value they placed on themselves in spending time with the other.

Both these relationships went on long-term, and seemed to make little impact on the addicted person. To *“Improved1”* the issue caused resentment, as if her worth was reduced to her ability to reduce drug consumption. The addicted participants developed paradoxical bonds where they and their sponsors were preoccupied with their own worth and the others’ behavior. The focus on the drug abuse became a mutual obsession that neither could depart from, despite the fact that the drug abuse rarely affected the sponsors directly. *“Improved2”* and her sponsor have multiple ruptures due to this tension, and only partially succeed in repairing the tares. The relationship is repaired to a point, but the sponsor confronts *“Improved2”* (again) about her level of intoxication during their meetings. To *“Improved2,”* who was a sensitive and private person, her sponsors’ demands were hurtful and her apologies were made too swiftly for *“Improved2”*s hurt to be healed:

At the time, I was happy she apologized, but in hindsight, she has told me that she felt very… I mean, she says to me that she “hadn’t expected such a resourceful person”. She had expected somebody in need of greater help. So then I asked her if she couldn’t get another person she could supporter in addition to me? And she quickly shifted the conversation over to something else. Like, “chop chop” (motions in air). So, it is kind of hard to receive an apology like that.

“Improved2” p. 11

An enmeshment that was slower to evolve in terms of confrontations was found between *“Unchanged1”* and her sponsor. *“Unchanged1”* was afraid of being rejected and reluctant to assert herself and her sponsor continued to reassure her for a long time. Over time, however, she became frustrated and like *“Improved2”*’s sponsor, started making demands to know more about the drug-use, and plans to get clean.

3. *Support perceived to be effective - mindful management of power imbalance:* in addition to supporting the addicted individuals in a practical way, the sponsors of all those who made improvement asked the addicted person for help, particularly in personal matters. *“Sponsor1”* involved with both *“Recovered1”* and *“Improved1”* perceived *“Recovered1”* to be more vulnerable in the relationship, and out of respect, maintained a distance. At a distance, however, it was difficult to be supportive. To help even out this power imbalance, he started asking *“Recovered1”* for favors:

4. *Taking control:* At the 2-year follow-up, only 2 addicted persons were in a process of “active recovery.” That is, they had maintained their reduced consumption and had no more than a minor slip in the past year. Additionally, they and others affirmed this status. Psychologically, the persons were different; *“Recovered1”* had an internal locus of control, believing himself capable of exerting a high level of influence on events affecting him. In the last year, however, he seemed to have lost control in defining his own identity due to financial problems. He had struggled with an additional stigma of mental illness most of his life, which was medicated. As such, he saw his emotional fluctuations as a normal part of himself and he was used to the idea that he chooses to self-medicate or regulate these fluctuations by the use of drugs.

“Sponsor1”: …he is a friend… and for me it is very natural that he sees me as one as well. I’d be very happy to be his friend… a friend means, you can talk about stuff, and you can ask for favors, small stuff. Actually, I have started doing that… I asked him for a favor… just to.. I mean, I didn’t have to ask about it… but I knew he could do it… so I asked, on purpose. To help build him up, let him give to me.

I: So, you give him your confidence, like that?

“Sponsor1”: Yeah, because often you feel like, no, you shouldn’t… hassle the other. But I mean that sometimes, it is actually the opposite. That by asking the other, you honor the other… if they want to give, then they can. That builds them up, they build their esteem and their confidence.

“Sponsor1” p. 36

*“Recovered2,”* in contrast, had an external locus of control, low problem and stigma awareness, and went from having a long-term pattern of heavy drinking, which he “denied to his children for years” (*“Recovered2”*, follow-up interview, *p*. 4), to acknowledge his problem and change behavior and identity, virtually overnight. The abrupt change was caused by a doubly concerning event: being caught for drunk driving when too intoxicated to recall driving, and having his adult children find out about it. To both *“Recovered1”* and “*Recovered2”* the responsibility for consumption seemed already internalized prior to the onset of our study.

For those who had recently improved, increased change-talk was observed after they had rejected their sponsors. In both cases, the individuals had struggled long-term with the feeling of not having their needs met in the relationship, and the process of rejecting the sponsors had a positive effect on the internalization of responsibility for using drugs. For *“Improved1”* the experience brought on the insight that he did not need a sponsor or “friend” *because* of his drinking problem. In fact, meeting his sponsor reminds him that he already has friends who are important to him.

I: Have you had any contact with your sponsor since the program?

“Improved1”: No, and we had talked about that, about having contact… and I said something like “that would be fine”… but that never happened. He has never initiated anything, and neither have I. And I don’t see a reason for it (laughs).

I: You are laughing (inquires)…

*“Improved1”: … I’m laughing, as it seems kind of comical to me… like, “never mind” maybe… lackadaisical in relation to…I mean, if I were to contact this person, whom I have gotten to know… I mean, this and that. Yeah, well, I laughed, because that is not the way people typically know me… people know me as pretty pleasing, you know? Almost to the point of self-destruction for the sake of caring for the other*… *and be kind and pleasant and good and right*, *in every way*… *but then this is a bit*. *I laughed*, *because it felt liberating to say that* “*no*, *there is no point to that”*, *kind of*… *(laughs)*

“*Improved1” p*. *17*

Both “*Improved1”* and his sponsor attribute the difficulty in finding something organic to do together to be the main reason why the relationship dissolved. They usually met in a café, but once “*Improved1”* decided he did not have to please his sponsor, the relationship lost its thrust in the recovery process. At the two-year follow up, and with increased self-esteem and continued progress, “*Improved1”* was on his way to building a new identity. As well as having a problem with alcohol, he now defined himself as traumatized in childhood, and he was exploring other interests by taking up new studies. At the 2-year follow up, his identity was shifting from someone afflicted by a condition that was like cancer “which is something you can sometimes manage, but not cure,” to the idea that he had been traumatized by abuse in his childhood, but chooses to drink when he gets scared. The relationships that were focused on the conversation itself did not impact the addicted individuals until they themself rejected the connection. Such rejections were made both by “*Improved1”* and “*Improved2”* late in the research study, who seemed enhanced in self-esteem by the events. For “*Improved3”* the internalizing of responsibility was not related to rejecting the sponsor, but to events where the sponsor asked for help.

“*It is such a wonderful feeling*. *You have the best conscience in the world; like you just got in the door after logging in the woods in the midst of winter*, *and you can finally relax*.*”*

*Improved3*, *p*. *23*

5. *Self-verification of positive identity:* Some participants (3) identified their addictions as disorders, a couple (2) did not identify with being disordered at all, defining themselves as living a “different” lifestyle. The remaining participants described themselves with a mix of stories including both disorder and lifestyle attributions. While we detected no difference in level of emotional distress between these groups at the onset of the study, the individuals labeled as “lifestyle users” did not seem to feel equally “out of control”, appearing more confident in their own control, and less worried about consequences related to their drug-use. They furthermore spoke of their using experiences with “plain speak”, comparing it to “normal”, and even socially condoned practices. One of the participants described being high on heroin like this:

As no participants were substance-free at the onset of the study, some (4) had encounters with their sponsors where they were forced to defend their decision or need to continue using drugs (or to use them with moderation), whilst continuing to benefit from the support program. These confrontations resulted in drop-out (1; mixed self-definition), failure to improve (2; “disordered self”), and firing of the sponsor (1; mixed self-definition). None of the “lifestyle users” reported of such experiences. While sponsors and others responded to the “lifestyle users” with suspicion at first, by the end of the study, two of them were nonetheless in the “improved” group. In fact, this group included two “disorder users”, two “lifestyle users”, and one user with a mixed definition. Members in this group all appeared to have more social opportunities, however, enabling them to present themselves as holding roles other than as “patient, or “user”. to present themselves to their sponsors as someone other than a “patient” or “user”, and other than someone in relationship with someone else. All had access to alternate social roles that they used to verify their self-worth throughout the recovery process. None of the participants appeared to be negatively affected by sponsors’ accepting a moderation approach to managing drug-use, and how negatively people were affected by sponsors’ confrontations depended on the availability of having alternate, positive identities to help obtain the sponsors’ respect.

## Discussion

In this study we set out to theorize on addiction recovery by examining social support dyads, and identifying factors that support positive long term changes. Fear of stigma and finding a common project made up the themes from the coding of baseline focus groups and interviews. By utilizing a case-study approach within a grounded theory design, we contrasted the processes of two improved groups of dyads including the addicted persons who were defined as “recovered” (*n* = 2) or “partially recovered” (*n* = 3) at the 2-year follow-up, with those of two unimproved groups including those “unchanged” (*n* = 3) and one person who had “dropped-out” (*n* = 1) . As such, the final codes of 1) identity supportive activity, 2) and 3) perceived to be ineffective- and effective support behaviors, , and 4taking control were contrasted on this axis from improved to unimproved, to reveal the final principle, self-verification of positive identity, and the positive identity model of change.

### Practical support is perceived to be effective

This study is a first to qualitatively examine the longitudinal effects of social support on addiction, and what we perceived to make up *effective support* was facilitated by focusing on something practical. The finding is consistent with research which has shown that one of sponsors’ main functions is to be communicators that provide compromise to the challenges that the recipients are faced with (Jacobson et al. [35]). It is also consistent with findings from research on “natural” addiction recovery, which has shown that it is the stabilizing aspects of relationships that are the most important elements in this process [11]. These findings bear resemblance to insights from the individual coping literature, which suggest that problem-focused strategies facilitate better health and life adjustment than emotion-focused coping [36].

### Being of use and taking control

We also found that taking responsibility for change was related to gaining social position in three ways; 1) by being able to help one’s own sponsor, 2) by being able to reject that person while acknowledged by others throughout and after this process had ended, and 3) by becoming a sponsor. In short, those who recovered had sponsors who invited them to be of use. The activities that grew out of the literal responses provided opportunities for role-reversals - situations where the addicted persons also were of use to the sponsors. These findings are consistent with that of a large randomized alcohol recovery study [37] (i.e., Project MATCH), where those helping or supporting others through AA were significantly less likely to relapse in the year following treatment, independent of the number of meetings they had attended. This study adds preliminary support to the act of helping, as opposed to the receiving of help, as a necessary function of recovery support relationships [38].

### Verification of positive self

On an individual level, participants with clearly developed identities related to “healthy” roles including being caretakers or having skills, were equally distraught by fear of stigma at the onset of the study as those who lacked such identity. Nonetheless, they appeared less negatively affected by events where they perceived themselves to be potentially judged by the sponsors, and fired their sponsors when they were unable to reflect a positive self-image. The finding is consistent with earlier work showing that in- versus out-group identification can moderate people’s tolerance for group demands when entering into AA [39]. It is also in support of principles of self-verification and self-enhancement theories, respectively, which purport that people seek social connections where existing schemas can be verified, and reject information that reduces the schema’s value [40]. As such, the findings of this investigation into recovery from substance abuse also fits within the traditions of symbolic interactionism – that people utilize symbols to predict how others will respond to them.

### Positive identity model of change

We perceived ineffective support to include the sponsor having a great deal of focus and concern on how much the participant was using, and a lack of practical support. Notwithstanding the high degree of emotional availability offered in these “ineffective” dyads, such dynamics created frustration with both parties, and was prevalent in the dyads that focused the activities on the relationship rather than on the individuals’ personal goals. “Effective support” was signified by attention to power imbalances by giving the less secure individual position to contribute to the relationship. Additionally, having meetings that were centered on supporting him or her somehow practically toward their personal goal were deemed to be crucial to recovery. Both contributing to the relationship and receiving practical support toward a personal goal are activities that are evidence of a person’s worth: one reflecting one’s efficacy, and the other showing evidence of one’s acceptability to another person. Other mechanisms are also possible including practical getting rid of problems that facilitate stability, as well as more time spent on non-drug related activities.

Taken together, the findings suggest that in order for support relationships to aid recovery, they have to be founded within a context that enables the person affected by addiction to build a positive self-image through mastery experiences that the support person *can* affirm. When interactions had occurred within the current study, people remained optimistic about their recovery if a positive self-representation also had been verified. In this case, it appeared as though fear of stigma, anxiety or paranoia also evoked by the meeting was attributed to “something about the sponsor”. As such, it is as if the two communicating partners must interact with or work through a third entity, namely the *project*, to ensure that self-verification of a *positive* self-concept indeed happens, and growth occurs. Conceptually, in treating people affected by addiction, helpers are not helping people overcome their addiction, but they are giving them an opportunity to show who they are. Furthermore, while we may technically be helping people build self-esteem, this wording is also misleading: the help we give is not an act of giving, but rather a process of receiving. Effective helpers are able to contain others’ negative emotions, and enable interactions that can evoke positive self-concepts. A mutually dignifying and shared understanding is an active force in recovery making.

**Figure 2:**
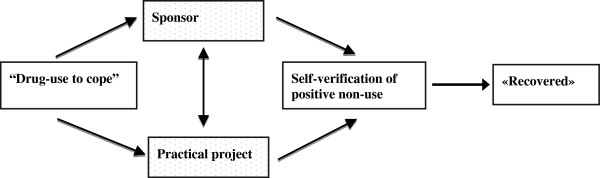
The positive identity model of change.

By focusing on social support, the the positive identity model supports the relevance of the recovery concept, and informs *why* it is relevant [15,16]. It offers a constructivist perspective on recovery, thus giving it a social-psychological content, and brings to the forefront its’ social, as opposed to biopharmacological etiology. In this study, recovery was possible when the partners together visualized and pursued a positive content. This outcome is fitting with insights from the hermeneutic philosophical tradition, where *true understanding* is considered possible only when two communicating parties focus on one another’s *content* rather than try to get into each other’s’ heads [41]. This because in order for the conversation to alter one’s thinking or concepts, we must first experience them as the other does, and therefore need to “fall into”, rather than conduct, our conversations. In the current study, having a practical project facilitated the mutual experiencing necessary for growth within the sponsoring relationship, and thus makes up the core component of what we call the *positive identity model of recovery* (see Figure 2).

### Limitations

The study was conducted with a small sample, using qualitative interviewing techniques as a single method to collect data. Also, while the addicted persons deemed recovered at the end were surely already motivated in such a way (e.g. already being enrolled in an educational program), that it affected the sponsoring relationship in such a way that an activity-based framing of it was obtained. Findings may therefore not generalize to addicted persons and sponsors in different settings. For example, in this study, the two dyads that developed the most clear pattern of enmeshment involved participants who were exclusively addicted to prescription drugs. It is possible that the use of prescription drugs is particularly challenging to sponsors, and future studies need to verify if this is the case. More broadly, however, further research should aim to validate the theoretical advancements across settings, with larger sample sizes, and explore the utility of the model when applied to marginalized individuals with difficulty articulating personal goals.

We were unable to get follow-up interviews for three addicted individuals believed to have relapsed. As a result, we were unable to verify our assessments of the cultural identities of these three addicted individuals. However, given the stability of such interpretations from baseline through follow up for the other subjects, and the fact that we were able to do indirect verifications through the key informant, we think it is unlikely that this has influenced the conclusions of the current study. Moreover, the combined longitudinal and contextual approach used in this study enabled observation of the long term interactions between sponsors and addicted persons. As such, the codes describe relational rather than subjective phenomena, which may have greater validity and prescriptive value as a general change model.

### Clinical implications

In this study, recovery ensued as part of a positive identity development indicated by activities that operationalized the roles of the sponsors and addicted individuals in such a way that they themselves came into position of giving support. Broadly speaking, the finding implies that recovery programs should entail opportunities for personally meaningful activities that can be socially shared. Future research should examine whether supportive relationships that offer practical support are indeed more effective in building self-esteem and positive recovered identity. This study has uncovered elements involved in the foundation for a social support trajectory towards recovery. Social support programs should perhaps not insist on constraining addicted persons to belong to recovery subcultures, but perhaps to a greater degree encourage people to design their personal recovery support, operationalized as something that promotes an already existing and healthier identity within themselves.

The findings inform ways to increase individual autonomy within support programs. Autonomy-supportive climates are typically understood as those that help people understand the rationale for treatment, acknowledge their feelings and perspectives, and emphasize personal choice [42,43]. Within professional treatment, which frequently emphasizes “talk therapy” as its method of delivery, motivational interviewing (MI), a client-centered and evidence-based counseling approach, supports autonomy by “rolling with resistance” and strengthening the client’s own verbalized motivations for change [44]. Sponsors are typically not trained to structure their conversations according to MI principles, however, making enmeshment an easy quagmire. Practical support may provide individuals with greater perceived control over their problems as it allows both parties to define the problem as at least partially, external to the person with addiction.

## Conclusion

Effective support was facilitated by ongoing availability by the sponsors, emotionally but more importantly, practically. The findings indicate that sponsors can be people who are non-users if the persons have the necessary skills to reduce social distance, and particularly if the user thrives on being “different”. Taken together, the findings give preliminary support toward a positive identity model of recovery support, indicating that the self-esteem gained from validation of a positive, non-using identity, relates with motivation for recovery. The study has exposed what may be a principle of addiction recovery - that mutual activity and a shared common goal may be criteria for utilizing social support in presence of personal and cultural differences.

## Competing interests

The authors declare they have no competing interests.

## Authors’ contributions

ABJ designed and carried out the study, as well as drafted the manuscript. HB co-analyzed the findings and edited the manuscript. FJD helped with the design and analyzes, and edited the manuscript. Finally, DKW helped with analyzes and edited the manuscript. All authors read and approved the final manuscript.

## Pre-publication history

The pre-publication history for this paper can be accessed here:

http://www.biomedcentral.com/1471-244X/13/201/prepub

## Supplementary Material

Additional file 1 Interview Protocol Topics by Group. Click here for file
