# Supplementary material for: Practical support aids addiction recovery: the positive identity model of change
Source: BMC Psychiatry. 2013 Jul 31;13:201. doi: 10.1186/1471-244X-13-201 (PMC3751355; doi:10.1186/1471-244X-13-201)
Supplement: Additional file 1 — Interview Protocol Topics by Group. [file 1471-244X-13-201-S1.docx]

**Additional file 1: Interview Protocol Topics by Group**

| **Addicts** | **Sponsors** | **Key Informant** | **Focus Group** | **Follow-up** |
| --- | --- | --- | --- | --- |
| History of drug use | General background | Program features (routines, regulations, etc) | How heard of program | Current pattern of use |
| Treatment efforts | Social network | Professional background | What motivated their enrollment | Events of milestones in the recovery work |
| General background | Experiences with the project and any previous sponsoring or treatment experience | Roles and tasks | How they experienced the matching process | Events or milestones within the relationship |
| Social network | Expectations and perceived quality of the relationship | Sponsors | What activities they pursued within their support dyad |  |
| Experiences with the project and any previous peer supporting or treatment experience | Activities and communication strategies | Participant recruitment | How the program had or could be of use |  |
| Expectations and perceived quality of the relationship | Events or milestones within the relationship | Selection and matching | How it could be improved |  |
| Activities and communication strategies | Handling privacy or boundaries | Follow-up | General worries, challenges, and surprises |  |
| Events or milestones within the relationship | Communication and follow-up with the program coordinator | Supervision |  |  |
| Handling privacy or boundaries | Contact with other sponsors or addicts | Events known to the coordinator (post) |  |  |
| Communication and follow-up with the program coordinator | Pperceived usefulness of the program and motivation for future participation | Sponsors and participant personal processes (post) |  |  |
| Contact with other sponsors or addicts |  | Meeting regularity (post) |  |  |
| Perceived usefulness of the program and motivation for future participation |  | pursued activities (post) |  |  |
|  |  | Experiences with potential follow-up meetings between the sponsors, participant, or dyad (post) |  |  |
